# Pseudo-Spectral Damping Reduction Factors for the Himalayan Region Considering Recorded Ground-Motion Data

**DOI:** 10.1371/journal.pone.0161137

**Published:** 2016-09-09

**Authors:** Anbazhagan P., Anjali Uday, Sayed S. R. Moustafa, Nassir S. N. Al-Arifi

**Affiliations:** 1 Department of Civil Engineering, Indian Institute of Science, Bangalore, India; 2 Geology and Geophysics Department, King Saud University, Riyadh, Saudi Arabia; 3 Seismology Department, National Research Institute of Astronomy and Geophysics, Cairo, Egypt; Beihang University, CHINA

## Abstract

Ground-motion prediction equations that are used to predict acceleration values are generally developed for a 5% viscous damping ratio. Special structures and structures that use damping devices may have damping ratios other than the conventionally used ratio of 5%. Hence, for such structures, the intensity measures predicted by conventional ground-motion prediction equations need to be converted to a particular level of damping using a damping reduction factor (DRF). DRF is the ratio of the spectral ordinate at 5% damping to the ordinate at a defined level of damping. In this study, the DRF has been defined using the spectral ordinate of pseudo-spectral acceleration and the effect of factors such as the duration of ground motion, magnitude, hypocenter distance, site classification, damping, and period are studied. In this study, an attempt has also been made to develop an empirical model for the DRF that is specifically applicable to the Himalayan region in terms of these predictor variables. A recorded earthquake with 410 horizontal motions was used, with data characterized by magnitudes ranging from 4 to 7.8 and hypocentral distances up to 520 km. The damping was varied from 0.5–30% and the period range considered was 0.02 to 10 s. The proposed model was compared and found to coincide well with models in the existing literature. The proposed model can be used to compute the DRF at any specific period, for any given value of predictor variables.

## Introduction

Most ground-motion prediction equations are available for a reference damping of 5%. The damping ratio depends on the structure, type of material, and ground shaking, among other characteristics [1]. In reality, however, structural systems may have a damping ratio other than 5%. Structures such as those used in power generating plants, dams, transmission and telecommunication facilities, and buildings with isolation systems are examples in which the damping values may not be 5% [2]. There is a need for a damping reduction factor (DRF) to convert the intensity measures at 5% damping as predicted by conventional ground-motion prediction equations, to other specified levels of damping. The DRF is generally estimated from various spectral ordinates like spectral displacement, spectral velocity, spectral acceleration, pseudo-spectral velocity, and pseudo-spectral acceleration (PSA). Many formulations have been proposed in the literature for DRF, although it is the initial work presented by Newmark and Hall [3] that has been adopted in the various international building codes. Most of the earlier studies used damping as the predictor variable for the estimation of DRF [4,5,6]. Damping ratio has also been combined with the period of vibration in some studies [7,8,9,10,11,12]. The spectrum amplification factors used by Newmark and Hall [3] are based on 28 accelerograms from nine earthquakes prior to 1973. These were from active seismic regions and had magnitude values between 5.3 and 7.5. The displacement response spectrum or pseudo-acceleration response spectrum were derived from these data. These factors are provided for the constant acceleration, velocity, and displacement domains. The model developed by Ashour [6] for a reduction factor in terms of the damping ratio alone was adopted by UBC (1994). In contrast, Wu and Hanson [12] derived the reduction factor from the displacement response, and formed a relationship in terms of both period and damping. This result has been implemented in the 1994 National Earthquake Hazards Reduction Program (NEHRP) for the design of buildings with passive energy dissipation systems. Naeim and Kircher [9] tabulated the mean standard deviation for the inverse of the reduction factor, while studying the influence of damping and period as well. NEHRP in 2000 made use of the two values proposed by Ramirez et al. [10] for periods equal to 0.2T_s_ and T_s_, where T_s_ is the period at the intersection of the constant velocity and constant acceleration regions [13]. In some studies [8,14], the effect of damping ratio, period, and site class on reduction factors derived from acceleration responses were investigated as well.

Lin et al. [13] have reviewed the existing models in terms of the mean and the dispersion of the reduction factor, and concluded that the values proposed by Newmark and Hall [3] are the smallest in short periods. Cameron and Green [15] tabulated the mean and the logarithmic standard deviations of the reduction factor from the displacement response. A sinusoidal excitation was used by Cameron and Green [15] to demonstrate the dependence on frequency content and duration. Stafford et al. [16] included the duration as an extra parameter in addition to the damping and period. Cardone et al. [17] reviewed the models from existing studies and found that the most accurate DRFs were those proposed by Wuand Hanson [12], and Lin et al. [8], which depend on both the damping ratio and the period of vibration. Hatzigeorgiou [18] developed a model in terms of damping and period alone, while Rezeian et al. [1] developed a reduction factor from the pseudo-acceleration response and formed a model in terms of moment magnitude, distance, and damping at specific periods.

A majority of the international codes have used the DRF to account for supplemental damping in the response of buildings. Newmark and Hall [3] is one of the pioneering works on DRF. The proposed model was accounted by the use of damping ratio in the building modeling. The current codes are based on a force-based design of structures, but the DRFs included are based on research conducted on the effect of damping on the displacement response of buildings [8]. Results from Lin et al. [8] show that there is a considerable difference between the DRF for acceleration, and that for displacement. The authors have also stated that if damping comes from the inelastic response of structures, i.e. because of the use of plastic hinges, then the design should be based on inertial force, and the reduction factors should be derived from acceleration response. This procedure is applicable to conventional buildings. It was also stated that if the damping is dominated by dissipation devices, then the design should be based on the restoring force (or displacement), and the reduction factor should be derived from the displacement response.

There are two approaches to estimate the DRF: (1) to develop prediction equations that directly estimate spectral ordinates at various levels of damping, and (2) to develop scaling factors to convert the ordinates to other levels of damping. Most of the existing studies have used predictor variables of time and damping ratio, although a few researchers [15,19] have managed to identify the effects of magnitude and distance on the reduction factor. The present study has explored the effect of site classification and added it to the variables of magnitude, distance, period, and damping ratio.

The Indian seismic code IS 1893 (2002) has adopted the second aforementioned method, and has provided the average response acceleration coefficient for a critical damping of 5% for rock, medium soil, and soft soil. A set of multiplication factors has been specified as a function of the damping ratio alone in order to scale the response parameters. The DRFs found in the Indian seismic design codes may be based on limited recorded data from India, and hence, does not necessarily represent the Indian scenario or knowledge database. The objective of this study, therefore, is to derive DRFs for the Himalayan region based on PSA, and to study the influence of parameters like damping, period, duration of ground motion, moment magnitude, hypocentral distance, and site classification. The DRF at β% damping used in this study is derived as follows:

$$DRF=\;\frac{PSA\;for\;\beta \%\;damping\;ratio}{PSA\;for\;5\%\;damping\;ratio}$$ (1)

An empirical model has also been developed for the prediction of the DRF in terms of these variables. In total, 410 horizontal accelerograms with a peak ground acceleration greater than 0.01 g, obtained from the Program for Excellence in Strong Motion Studies (PESMOS) at the Indian Institute of Technology, Roorkee has been used in this study. The data range across site classes A, B, and C with magnitudes ranging from 4 to 7.8, and hypocentral distances up to 500 km. The site classification set in Mittal et al. [20] has been adopted, and parameterized values of 4, 3, and 2 were used corresponding to site classes A, B, and C, respectively. The damping ratio (ξ) has been varied in the range of 0.5–30%, with the period in the range of 0.02–10 s.

## Strong Motion Database

**Fig 1:**
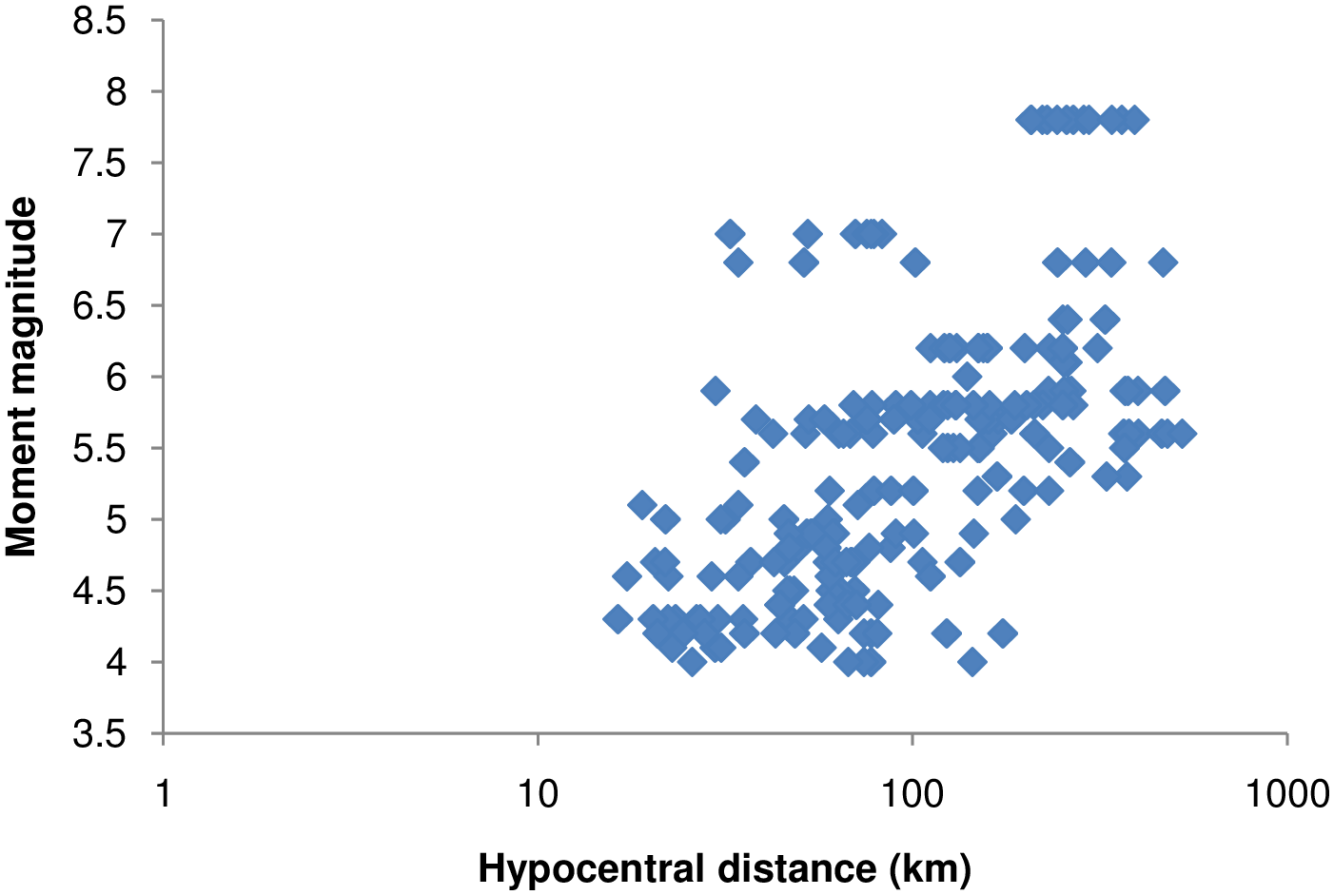
Magnitude—distance distribution of the horizontal components of the data used.

**Fig 2:**
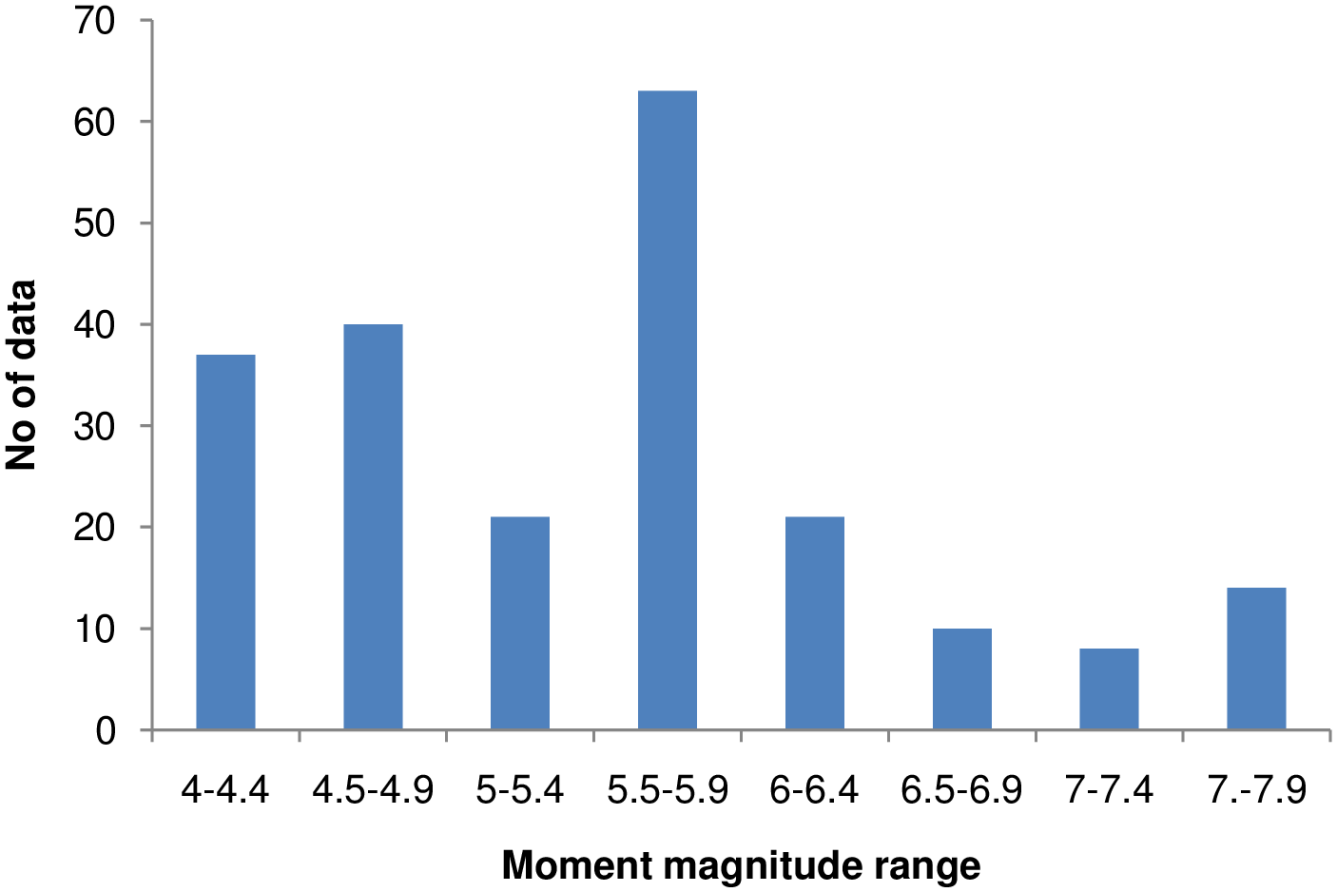
Distribution of data with respect to moment magnitude.

**Fig 3:**
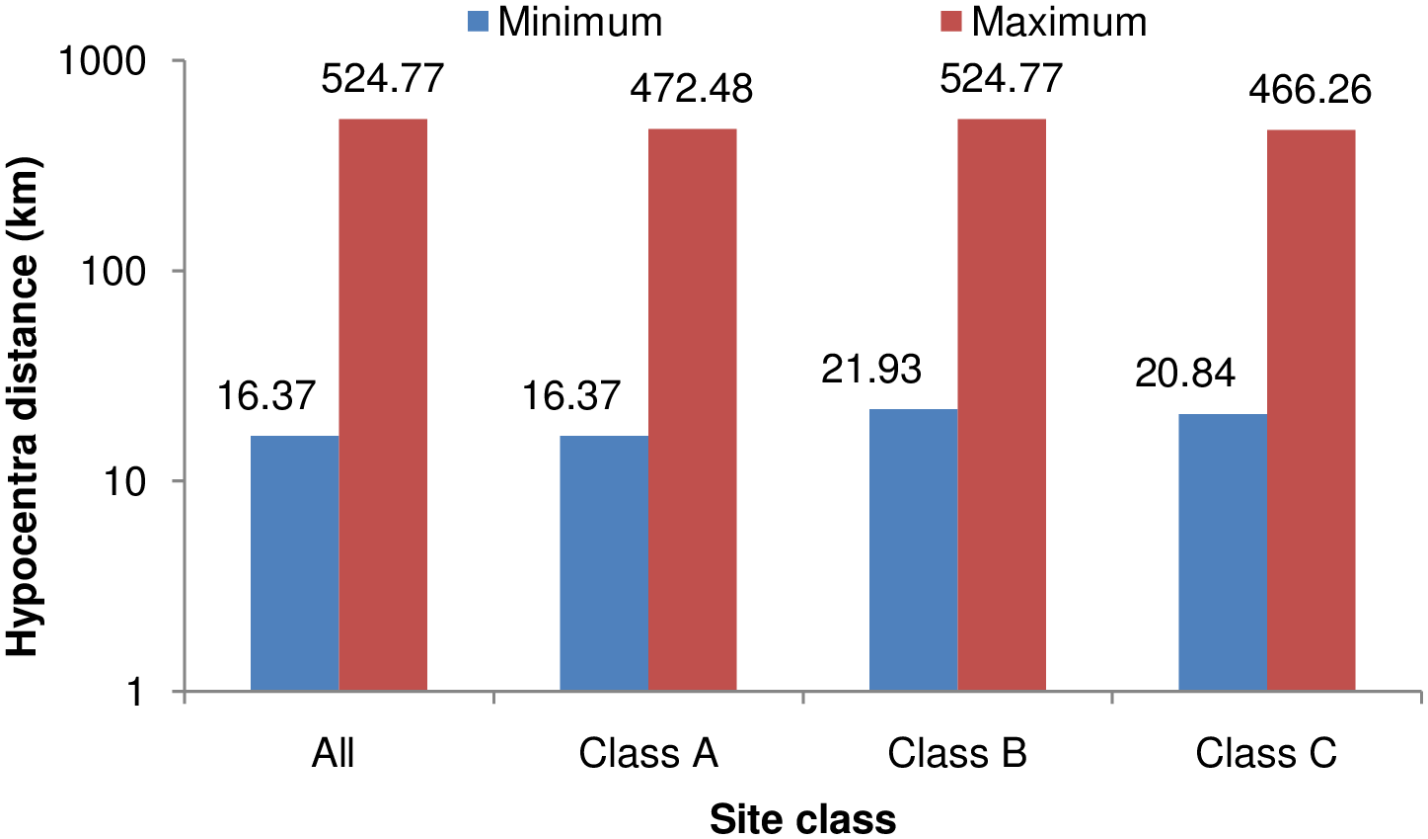
Range of hypocentral distance over the site classes.

**Fig 4:**
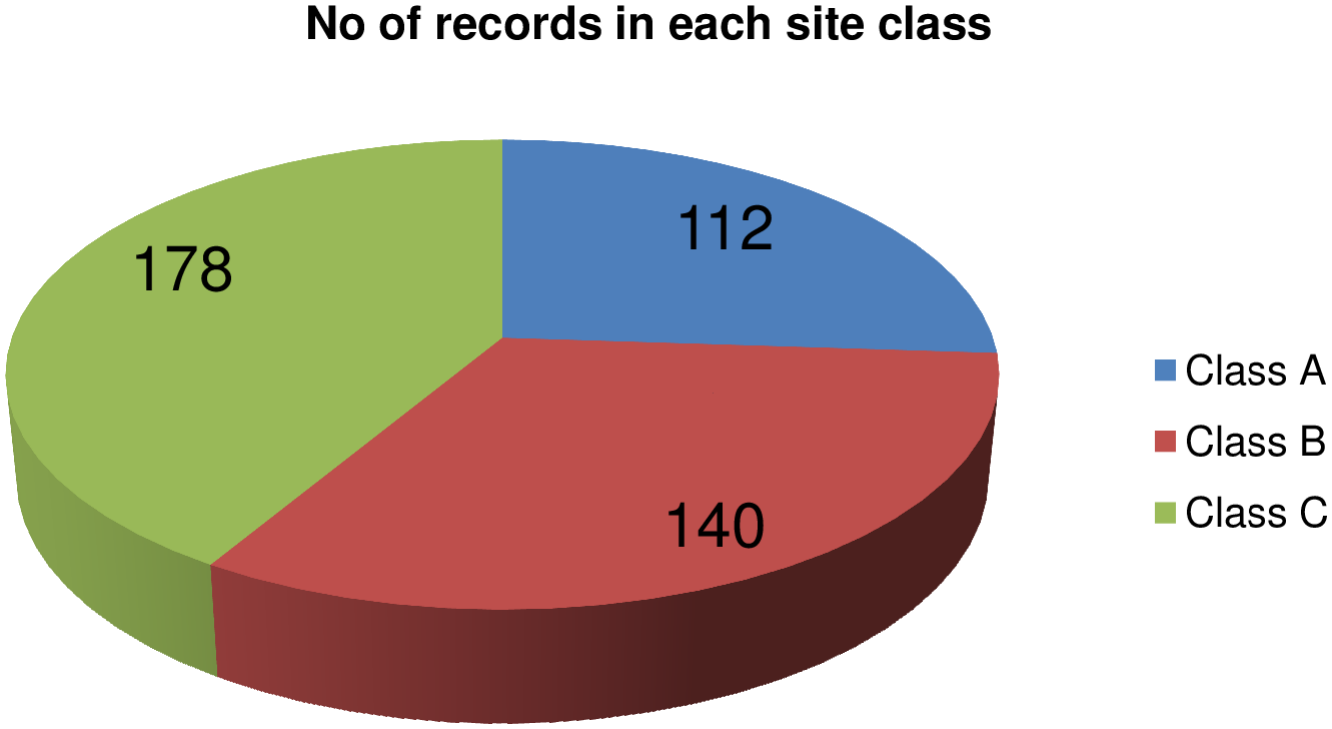
Data distribution among different soil types.

The recorded earthquake ground-motion data were obtained from the PESMOS at the Department of Earthquake Engineering, Indian Institute of Technology, Roorkee, India. The Department of Earthquake Engineering operates a network of about 300 strong motion accelerographs in the northern and northeastern parts of the country, which covers the entire Himalayan region. The network covers part of Himachal Pradesh, Punjab, Haryana, Uttaranchal, Uttar Pradesh, Bihar, West Bengal, Sikkim, and northeastern India. These stations are located in seismic zones V and IV, and include some heavily populated cities within seismic zone III as per the Indian seismic code IS:1893 (2002), all within the Himalayan region [21]. The Himalayan region was selected for study because the area had been subjected to many devastating earthquakes in the past. Baseline correction and filtering (low pass filter) was applied to the data by the PESMOS before dissemination. A selected number of 410 accelerograms recorded between 1986 and 2015 were used to develop the model for the DRF using regression analysis. An earthquake with moment magnitudes of ground motions greater than 4 and peak ground acceleration >0.01 g was chosen for the study. Instrumented sites were classified considering soil types and physical descriptions of the near surface materials according to Mittal et al. [20]. More information about the instrumentation and site classification can be obtained from Mittal et al. [20] and Kumar et al. [21]. The DRFs were calculated individually for the two components at different damping and periods as per Eq 1. These values were combined into an average value at different damping and periods, and were considered as the factor for that particular ground motion. The magnitude—distance distribution of the data can be observed from Fig 1. The number of data points used for the study in each magnitude range is shown in Fig 2. It can be noted that the majority of the data possessed moment magnitudes in the range 4–6, while the remaining M_w_ > 6. Fig 3 shows the minimum and maximum hypocentral distances for different site classes. Fig 3 shows that the lowest hypocentral distance is 16.37 km for site class A, while the maximum is 524.76 km for site class B. Site class A data was available up to 472.48 km, while site class C data was available up to 466.26 km. The number of data points belonging to different site classes, grouped as in Mittal et al. [20], is shown in Fig 4. It can be seen from Fig 4 that the data set is dominated by records obtained at site class C, followed by site class A, with the lowest number of records belonged to site class B. The variables used in this study are moment magnitude M_w_, hypocentral distance R (km), and site classification S. Moment magnitude has been used since it tied the magnitude directly to earthquake source processes, and does not saturate for magnitudes greater than 6. It is also directly proportional to the area of the fault plane that ruptured multiplied by the average displacement along the rupture plane [22]. Path parameters (hypocentral distance) are used to represent the spread of the seismic energy from the earthquake source to the site of interest. Based on the composition of the subsurface, different locations at the same distance from the causative fault will experience different durations during earthquakes. The influence of site classification was represented by parameterized values adopted based on the site class.

## Predictor Variables Used

**Fig 5:**
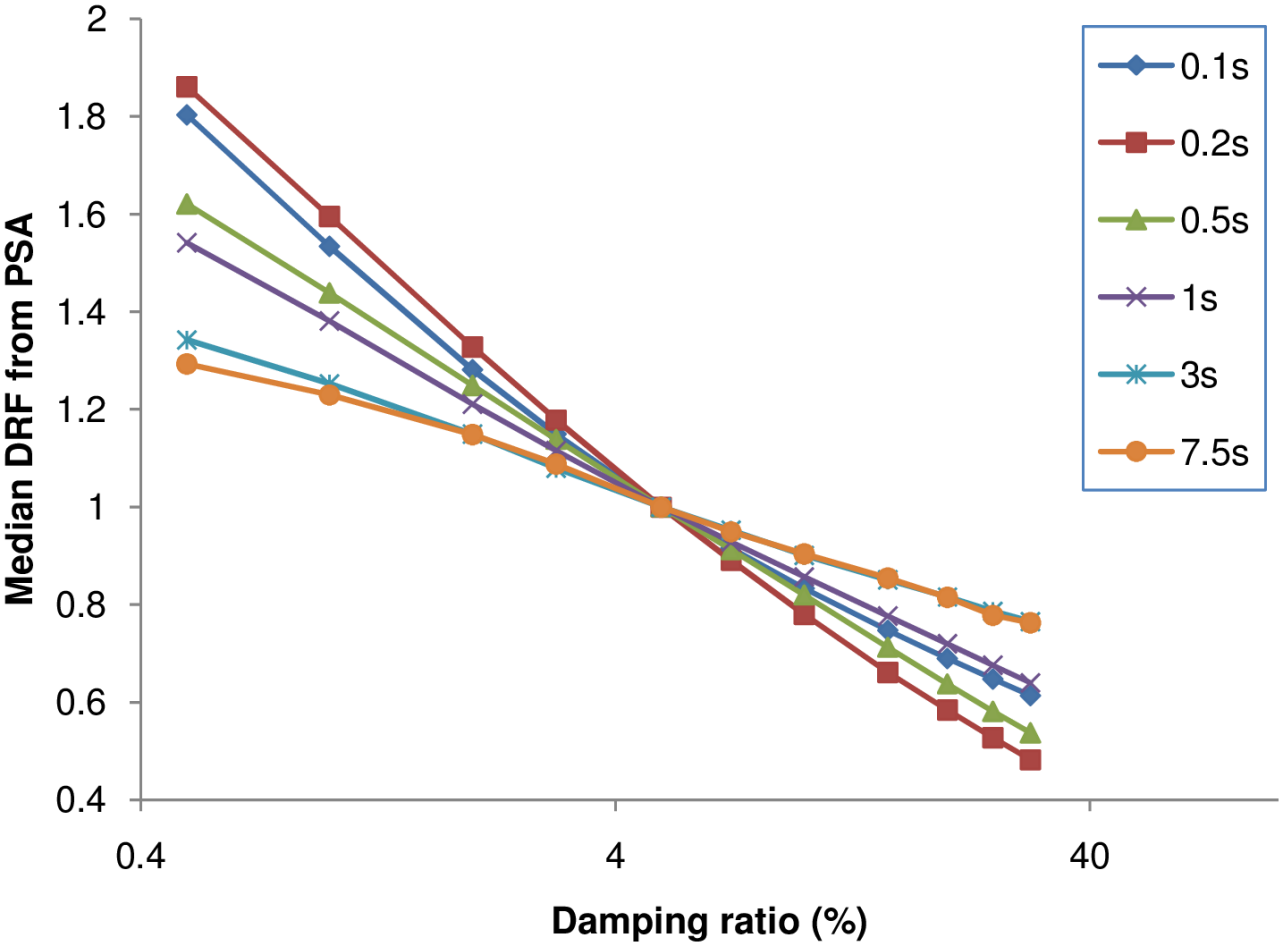
Influence of spectral period and damping on DRF computed from PSA.

The variables that might influence the DRF, and the trends observed between the variables and the DRF, are explained. The findings are then used to identify the potential predictor variables for the correlation. As mentioned earlier, damping ratio and spectral period are the principal variables, and these can be understood from the definition of DRF and the relation between PSA and period. The influence of damping and period on the DRF can be observed in Fig 5. The median DRF for each record, computed from the PSA ratio, was plotted as a function of the damping ratio in this figure. It can be seen that the DRF obtained from the PSA varies from 1.86 to 0.4 when the damping ratio increases from 0.5 to 30%.

**Fig 6:**
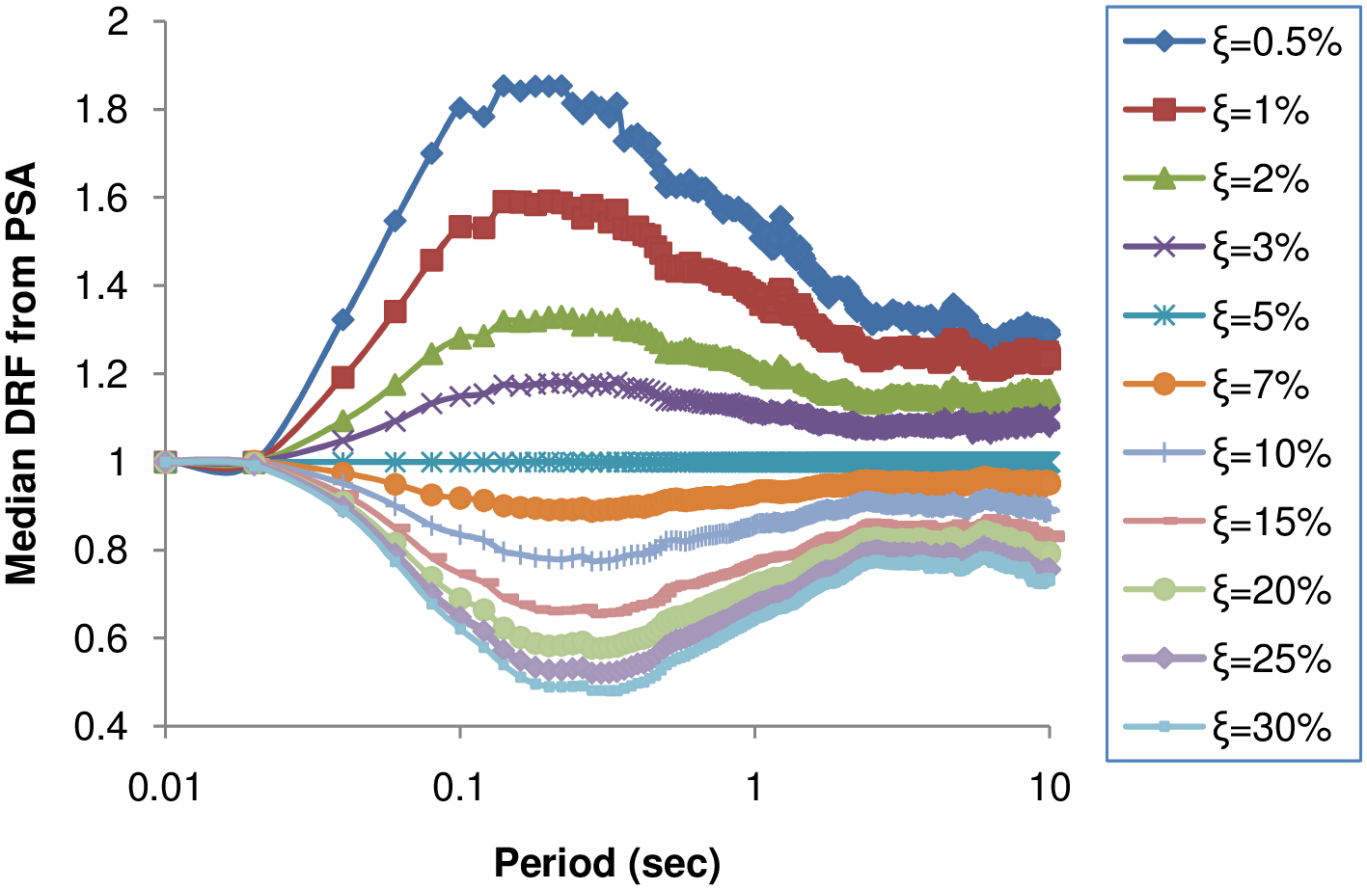
Influence of spectral period and damping on DRF computed from PSA.

The values of DRF have been plotted as a function of period at various levels of damping in Fig 6. The DRF values tend to become unity at lower and higher periods because the forces in a very stiff or very flexible structure are relatively independent of the damping ratio [1]. This trend is observed in the DRF derived from the PSA irrespective of period and damping. Almost no dependence on period is seen between 0.15–0.5 s for the DRF from the PSA at damping greater than 2%, but there is a strong dependence as DRF approaches unity for very low and very high periods. The variation of DRF with period is more at damping ratios less than 1% as suggested in Rezeian et al. [1].

**Fig 7:**
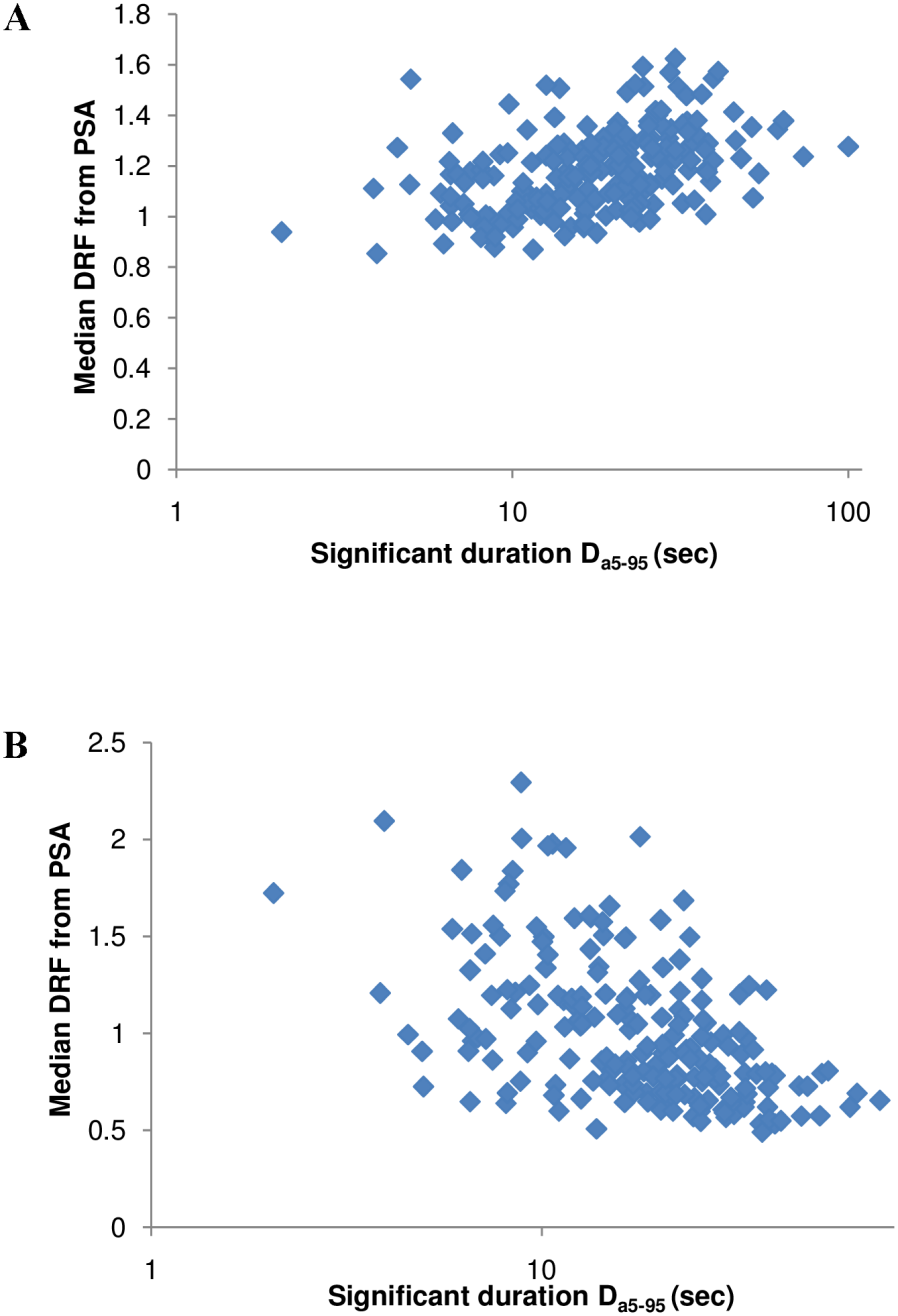
a–b. The influence of significant duration on median DRF at 2% and 20% damping.

Many researchers [15,16,19] have included strong ground-motion duration as an important factor affecting the DRF. Fig 7a and 7b shows the graph between median values of the DRF and significant duration D_a5–95%_ at damping ratios of 2, 20%, and a period of 1 s. An increasing trend is observed when the duration for damping is 2%, whereas a decreasing trend is observed at higher damping. The effect of duration on the DRF cannot be used in practice since duration is not of importance in the current design scenario. Hence, the influence of duration on DRF was captured by the inclusion of magnitude, distance, and site class in the current model. A strong positive correlation between duration and magnitude, and a moderate correlation with distance, is expected [23].

**Fig 8:**
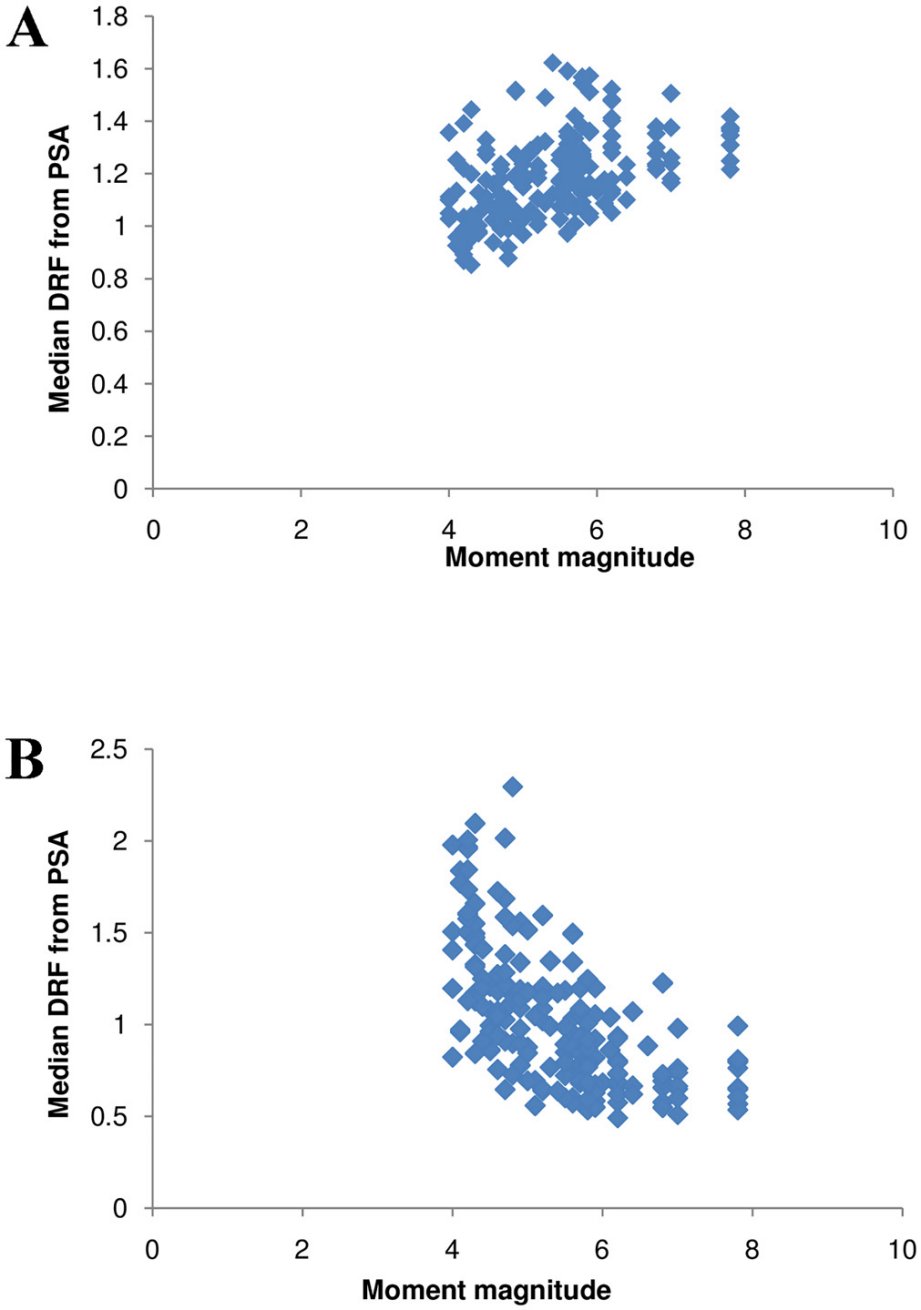
a–b. The influence of magnitude on median DRF at 2% and 20% damping.

**Fig 9:**
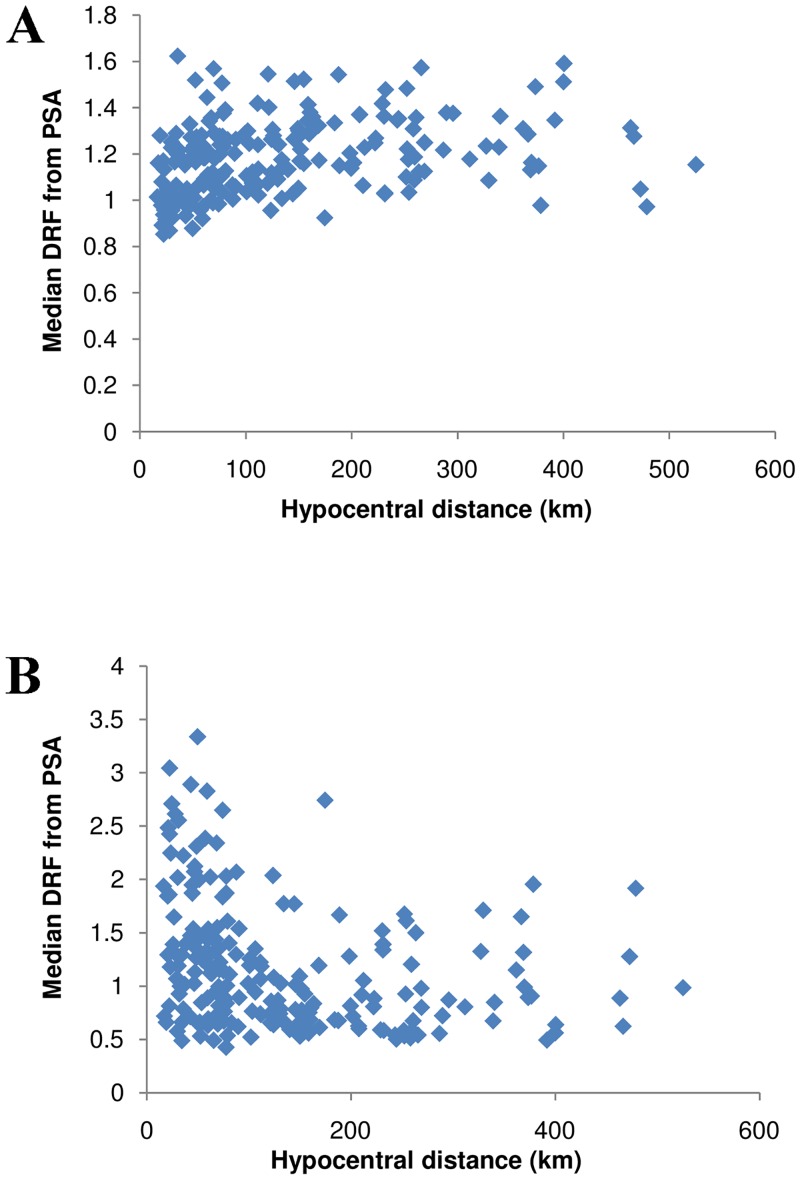
a–b. The influence of hypocentral distance on median DRF at 2% and 20% damping.

A significant dependence between the DRF obtained from the PSA and the moment magnitude M can be observed in Fig 8a and 8b. The figure is for T = 1 s, along with a fitted line to capture the trend in the data. Less significant patterns between the DRF and the hypocentral distance can be observed in Fig 9a and 9b. As observed for the duration, opposite trends were evident between the DRF and the magnitude, as well as the distance for both low and high damping ratios. At low damping, the DRF was found to increase with distance, which might be due to the effect of ground-motion duration [2]. The relation of DRF with duration, magnitude, and distance coincides with the conclusions of existing studies. A linear magnitude term is found to be necessary to capture the dependence on M. The addition of a logarithmic term for hypocentral distance and a linear term for site classification, as for the duration parameter, would be sufficient.

## Model Development

In this study, the general form of the equation to be used is as follows,

ln(DSF)=μ(β, T, source, site, path;b) (2)

where *μ* represents the mean of ln(DSF) and is a function of damping ratio β, spectral period T, source, site and path parameters such as moment magnitude, hypocentral distance, and site classification; b is the vector of regression coefficients.

The DRF model was systematically built by trying various functions of the key predictor variables influencing the damping scaling. First, DRF was calculated for the R < 520 km records. For each combination of the 22 specified periods and 11 damping ratios, the data were analyzed and the dependence of the DRF on potential predictor variables (e.g., duration, magnitude, distance, and site conditions) was investigated. Visual inspection of the plotted data revealed a strong logarithmic dependence of the DRF on duration and a strong possibly linear or quadratic dependence on the magnitude (stronger at longer periods), a weaker but noticeable dependence on distance, and negligible dependence on soil conditions. In order to capture the dependence on duration while including magnitude and distance in the model, ln (DRF) was regressed on the predictor variables **M** and R at specified T and damping.

For each combination of damping and period, the factors were regressed with magnitude, hypocentral distance, and site classification. The primary regression coefficients were then regressed with the damping ratio to find the final coefficients.

Multiple linear step regression was carried out, and the final model was of the form:

$$lnDRF=b_{0}+b_{1}\text{ln}\left(\xi \right)+b_{2}{\left(\text{ln}\left(\xi \right)\right)}^{2}+\left[b_{3}+b_{4}\text{ln}\left(\xi \right)+b_{5}{\left(\text{ln}\left(\xi \right)\right)}^{2}\right].M+\left[b_{6}+b_{7}\text{ln}\left(\xi \right)+b_{8}{\left(\text{ln}\left(\xi \right)\right)}^{2}\right].\text{ln}\left(R\right)+\left[b_{9}+b_{10}\text{ln}\left(\xi \right)+b_{11}{\left(\text{ln}\left(\xi \right)\right)}^{2}\right].S+Є$$ (3)

where *ξ* is the percentage damping ratio and b_i_, i = 0, 1….11 are the regression coefficients, which are listed in Table 1 for each specified T. For deriving the regression coefficient, multi-linear regression analysis has been used. The vector of all the independent parameters were regressed with the dependent variable (*lnDRF*) using least square analysis, which minimizes the sum of the squares of the errors made in the results. Based on that non-dependency of error on the predictor values, this method calculates the unbiased estimators. Further, based on the analysis, the error term (Є) was calculated, and is shown in Table 1. A similar procedure has been adopted for all the periods, and the regression coefficients corresponding to each independent parameter for different time periods are given in Table 1.

**Table 1:** The regression coefficients for the model for DRF computed from PSA.

T(s)	b_0_	b_1_	b_2_	b_3_	b_4_	b_5_	b_6_	b_7_	b_8_	b_9_	b_10_	b_11_
0.02	0.0264	-0.0098	-0.0040	-0.0036	0.0027	-0.0004	-0.0007	-0.0007	0.0008	-0.0016	0.0004	0.0004
0.04	0.5882	-0.3385	-0.0151	-0.0593	0.0341	0.0005	-0.0089	0.0010	0.0036	-0.0222	0.0121	0.0013
0.06	0.4738	-0.1822	-0.0724	-0.0127	-0.0080	0.0095	-0.0216	0.0087	0.0034	-0.0100	0.0031	0.0030
0.08	0.3285	-0.0479	-0.0973	0.0024	-0.0157	0.0080	0.0055	-0.0150	0.0087	-0.0025	-0.0035	0.0029
0.10	0.3531	-0.0964	-0.0739	0.0172	-0.0250	0.0075	0.0015	-0.0078	0.0059	-0.0152	0.0137	-0.0027
0.14	0.3229	-0.0905	-0.0693	0.0462	-0.0464	0.0111	-0.0297	0.0245	-0.0033	0.0030	-0.0005	-0.0010
0.20	0.0335	0.0843	-0.0737	0.0547	-0.0415	0.0064	0.0229	-0.0168	0.0013	0.0069	0.0004	-0.0024
0.24	0.0361	0.0176	-0.0264	0.0380	-0.0250	0.0002	0.0374	-0.0211	0.0003	0.0110	0.0010	-0.0056
0.30	-0.0362	0.0688	-0.0288	0.0549	-0.0393	0.0035	0.0415	-0.0174	-0.0052	-0.0050	0.0073	-0.0035
0.34	0.0045	0.0367	-0.0264	0.0617	-0.0453	0.0038	0.0381	-0.0125	-0.0061	-0.0260	0.0224	-0.0036
0.40	0.0781	-0.0300	-0.0135	0.0486	-0.0312	0.0004	0.0323	-0.0101	-0.0054	-0.0269	0.0210	-0.0026
0.44	-0.0110	0.0387	-0.0207	0.0388	-0.0219	-0.0022	0.0404	-0.0193	-0.0023	0.0067	-0.0055	0.0007
0.50	0.0329	0.0073	-0.0168	0.0691	-0.0361	-0.0038	0.0039	-0.0010	-0.0007	-0.0252	0.0132	0.0003
0.75	-0.1088	0.0557	0.0073	0.0987	-0.0544	-0.0031	-0.0061	0.0051	-0.0013	-0.0230	0.0204	-0.0049
1.00	-0.1073	0.0205	0.0329	0.0900	-0.0402	-0.0103	0.0046	-0.0057	0.0022	-0.0348	0.0257	-0.0033
1.50	-0.1690	0.0473	0.0431	0.1026	-0.0470	-0.0106	0.0003	-0.0001	-0.0001	-0.0480	0.0304	-0.0018
2	-0.3093	0.1473	0.0197	0.1484	-0.0719	-0.0112	-0.0388	0.0169	0.0049	-0.0335	0.0192	0.0010
3	-0.1996	-0.0025	0.0556	0.1041	-0.0351	-0.0166	-0.0076	-0.0098	0.0091	-0.0275	0.0243	-0.0014
4	-0.1935	0.0706	0.0362	0.0962	-0.0332	-0.0182	-0.0178	-0.0007	0.0080	-0.0177	0.0061	0.0035
5	-0.1347	0.0297	0.0288	0.0665	-0.0173	-0.0143	0.0018	-0.0070	0.0052	-0.0088	0.0012	0.0031
7.5	-0.1347	0.0297	0.0288	0.0665	-0.0173	-0.0143	0.0018	-0.0070	0.0052	-0.0088	0.0012	0.0031
10	0.2399	-0.1409	-0.0080	-0.0359	0.0204	-0.0004	0.0379	-0.0141	-0.0029	-0.0070	0.0037	-0.0001

**Fig 10:**
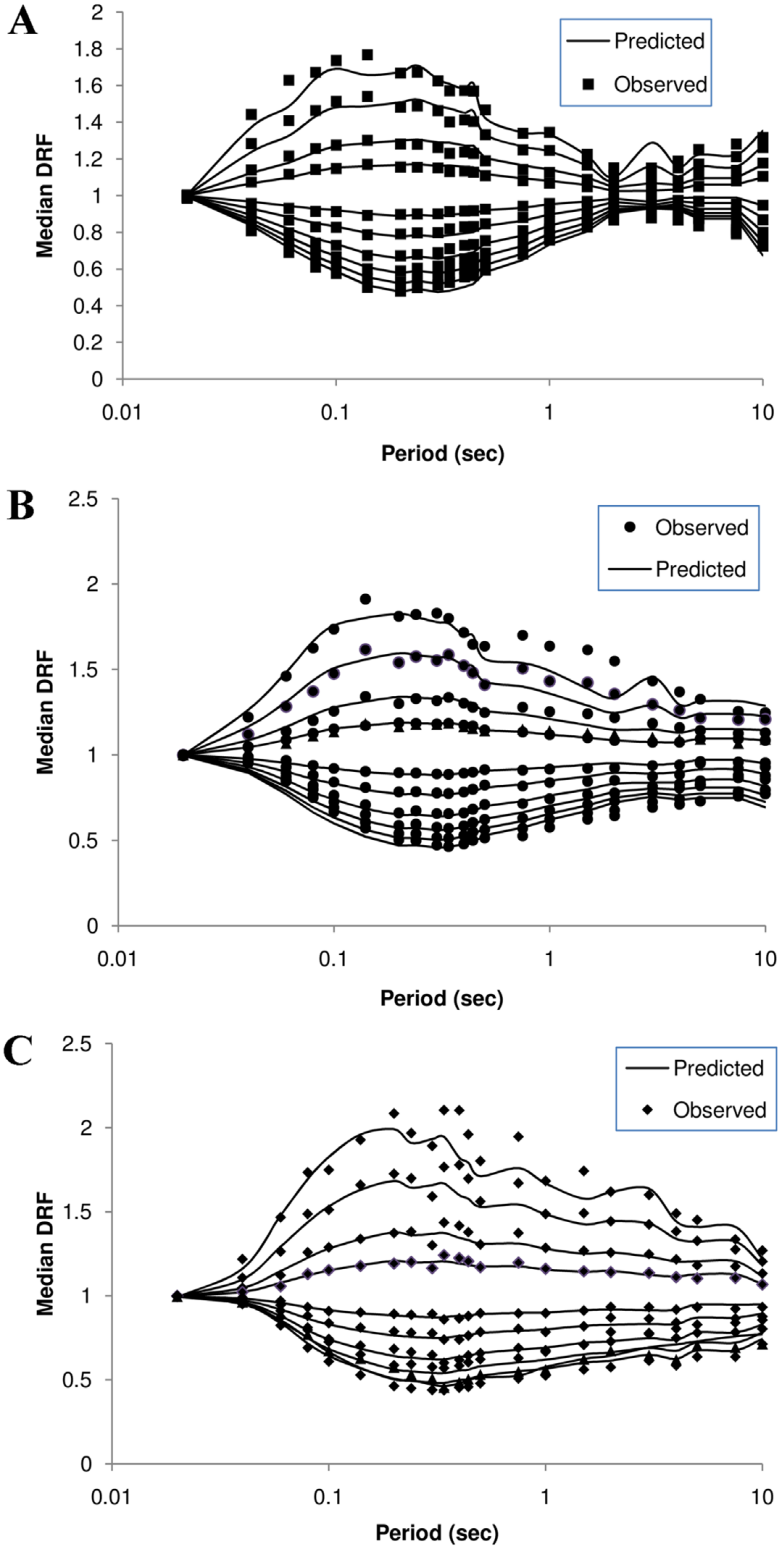
a–c. Data binned for M and R superimposed on plots of proposed model for M = 4.5, 5.5, and 6.5 at R = 125 km.

Since the proposed model is empirical, a close agreement between the model and the data should be expected. To visually validate the model against the data, plots similar to Fig 10 were generated, in which the predicted mean DRF is plotted for M = 4.5, 5.5, and 6.5 at a hypocentral distance of 125 km. Good agreement is seen between the data and the model. The minor variations found might be due to the wide magnitude and distance bins utilized.

## Results and Discussion

Lin et al. [13] emphasized that the DRF is usually needed in two cases. One is for the design and analysis of structures with added passive energy dissipation systems (damping devices) and isolation systems (NEHRP 2000, UBC 1997, FEMA-273 1997, IBC 2000). Since these structures always have high damping ratios, the DRF accounts for the effect of supplemental damping on the force and displacement responses of such structures. The second case is for predicting the maximum displacement demands of an inelastic structure from the maximum displacement demands of an equivalent linear system [13]. A visual comparison was made between the predicted model and the selected existing models. Models that have used similar data and applicability range of predictor variables were selected for comparison.

**Fig 11:**
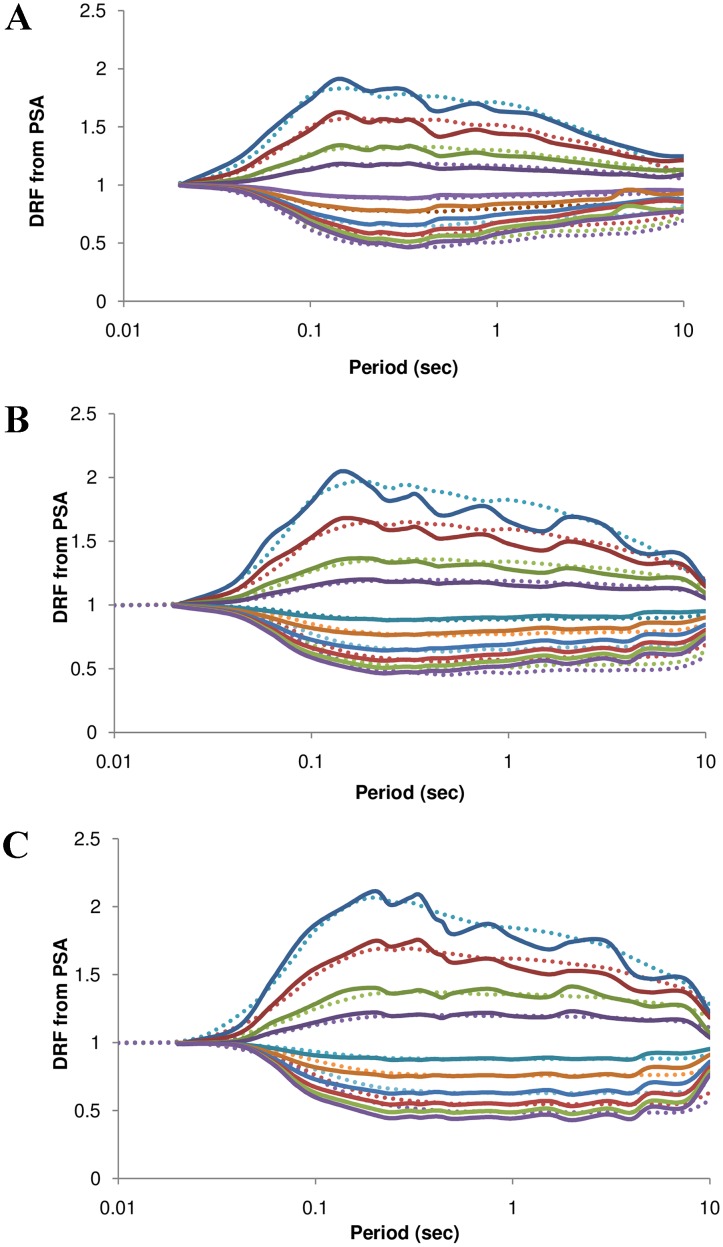
a–c. Comparison between the proposed model and model developed by Rezeian et al. [1] for magnitudes 5.5, 6.5, and 7.5 at a distance of 200 km. The solid lines represent the proposed model and dotted lines denote the model developed in Rezeian et al. [1].

Similar models that used source, site parameters, damping, and period are selected for comparison with the proposed model. The model developed by Rezeian et al. [1] was compared with the model proposed in Fig 11a–11c for the magnitudes 5.5, 6.5, and 7.5, and a distance of 200 km. 2250 records with magnitudes in the range 4.2–7.9 were used. A model was developed for the DRF from the PSA as a function of magnitude, distance, and damping ratio at specified periods. The model was valid for periods from 0.01 to 10 s and for damping levels varying between 0.5 and 30%. As shown in Fig 11, it is clear that the models were similar up to a period of 0.5 s for the magnitudes 5.5 and 6.5. The proposed model gives higher values for periods greater than 0.5 s. For higher magnitudes, the proposed model matches with the model in Rezeian et al. [1] for a damping ratio of less than 5%. For higher damping values, the developed model gives lower values when compared to the existing model.

There are no other studies that have considered both the effect of magnitude and distance in a model. Therefore, for the sake of comparison, models that have included the damping ratio and period have been selected and presented as supporting information. In addition to direct comparison of proposed model with recent study, proposed models are compared with models that have included the damping ratio and period and presented as Supporting Information. Newmark and Hall [3] is one of the pioneering works that proposed values for the DRF, using data from 28 accelerograms. It can be observed from S1 Fig that the peak values are similar at different periods at lower magnitudes. The values are similar at higher damping levels for all magnitudes.

Idriss [11] has defined adjustment factors normalized with respect to 5% damping using two sets of expressions, which are similar to Newmark and Hall [3]. One set of expressions is valid for damping ratios less than 5%, and the other for ratios greater than 5%. The expressions are applicable for the range 0.03–5 s and damping ratios of 1, 2, 3, 5, 7, 10, and 15%. The set of expressions is a function of the period and damping ratio only. The comparison between the proposed model and the model in Idriss [11] can be seen in S2 Fig

Therefore, based on the results of a regression analysis and comparison, it can be concluded that the proposed models are capable of predicting DRF values for the Himalayan region with high accuracy. Given the magnitude, distance, and the site class of a region, the reduction factor can be calculated for a particular value of damping at any specific period. The DRF derived from the displacement response (or pseudo-acceleration response) can be used for the design of buildings with damping devices.

## Summary

In the present study, 206 horizontal acceleration time histories that were recorded until 2014 were used to develop an empirical relationship to estimate the DRF. The proposed relationship can be used to convert the PSA values at 5% damping to the PSA values of other damping values. The effect of parameters like moment magnitude, hypocentral distance, site classification, duration of ground motion, damping, and period was studied. Duration was found to be a parameter that strongly affects the reduction factor; however, since its usage is not ideal, the dependence is captured through the inclusion of magnitude, distance, and site classification, all of which affect duration. Regression analysis was carried out on reduction factors at 22 specific periods and 11 damping ratios. The dependence on magnitude, distance, and site classification was captured using a linear term and a logarithmic term. The proposed empirical equation was compared with computed values from the data, and with the existing literature. The model was found to have good predictive performance, and hence could be used for the prediction of the DRF in buildings with added dissipation devices. The model is applicable for damping ratios from 0.5 to 30%, magnitudes from 4 to 7.8, and distances less than 520 km.

### Data and Resources

The processed ground-motion parameters for earthquakes before 2005 were obtained from the Strong-motion Virtual Data Center—which was developed by the University of California, Santa Barbara—and incorporated as a part of the Centre for Engineering Strong Motion Data at http://strongmotioncenter.org/vdc (last accessed August 2015). From the 192 ground-motion recordings, 124 were collected from the strong motion instrumentation network of Indian Institute of Technology, Roorkee that covers the Indian Himalayan range from Jammu and Kashmir to Meghalaya. This data is freely available and downloaded from the website http://www.pesmos.in (last accessed August 2015).

## Supporting Information

S1 Fig Comparison between the proposed model and the model developed by Newmark and Hall [3] for magnitudes 5.5, 6.5, and 7.5 at a distance of 200 km. The solid lines represent the proposed model and dotted lines denote the model developed in Newmark and Hall [3].
(PDF)

S2 Fig Comparison of the proposed model for magnitudes of 5.5, 6.5, and 7.5 for a hypocentral distance of 200 km with the model proposed by [11]. The solid lines represent the proposed model, while the dotted lines represent the model developed by Idriss [11].
(PDF)
