# Supplementary figures and images for: Pseudo-Spectral Damping Reduction Factors for the Himalayan Region Considering Recorded Ground-Motion Data
Source: PLoS One. 2016 Sep 9;11(9):e0161137. doi: 10.1371/journal.pone.0161137 (PMC5017774; doi:10.1371/journal.pone.0161137)

DRF from PSA

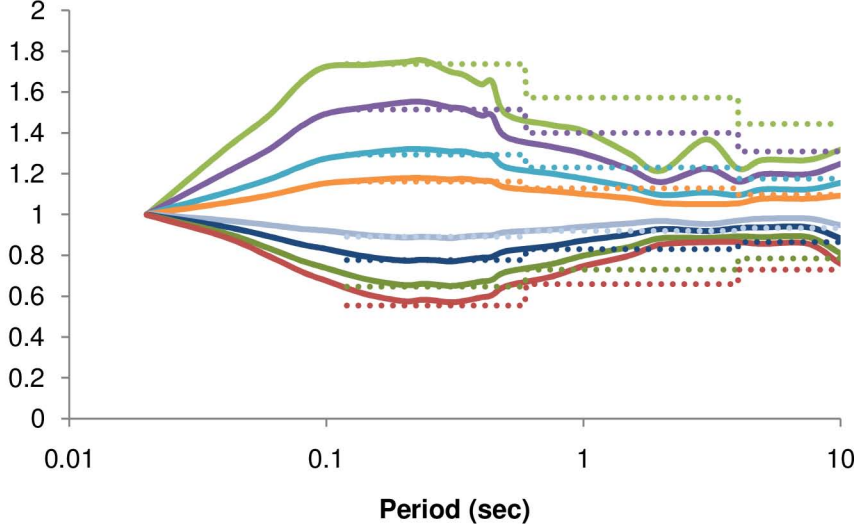

DRF from PSA

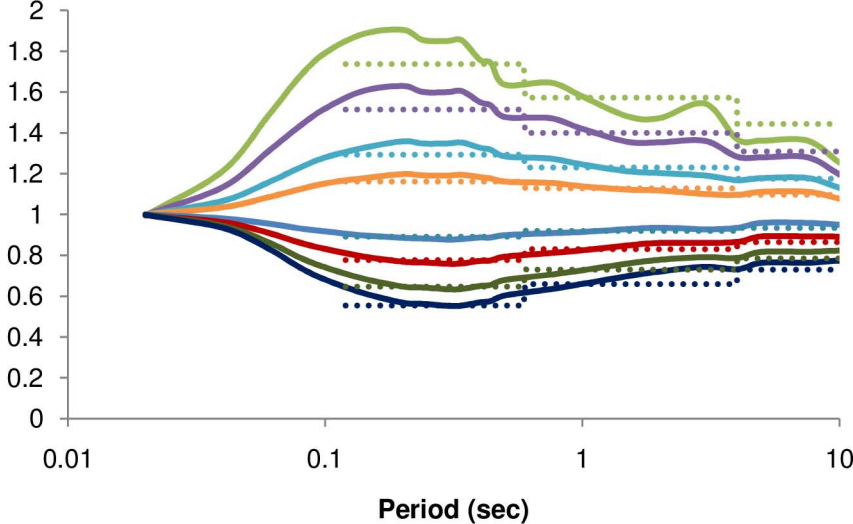

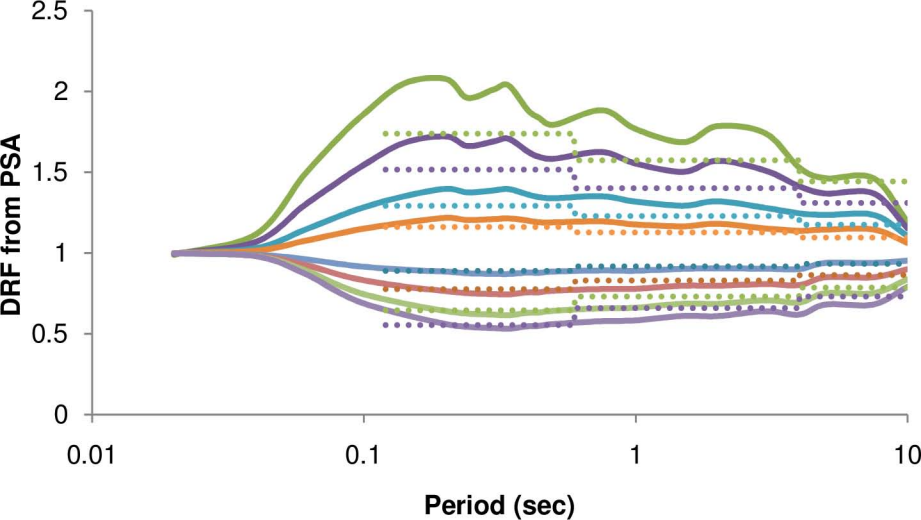

Supplement: S1 Fig — The solid lines represent the proposed model and dotted lines denote the model developed in Newmark and Hall [3]. (PDF) [file pone.0161137.s001.pdf]

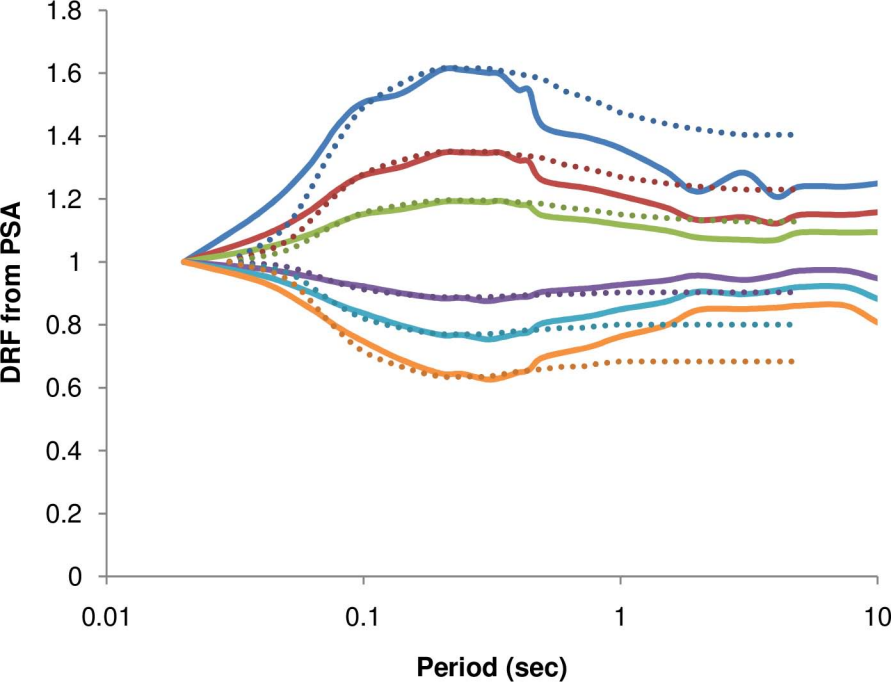

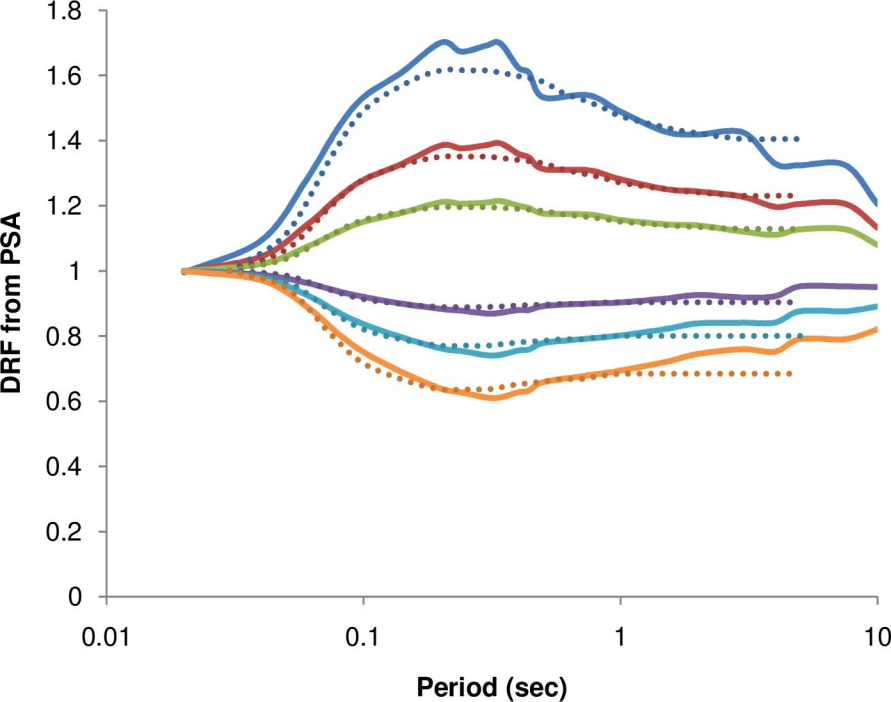

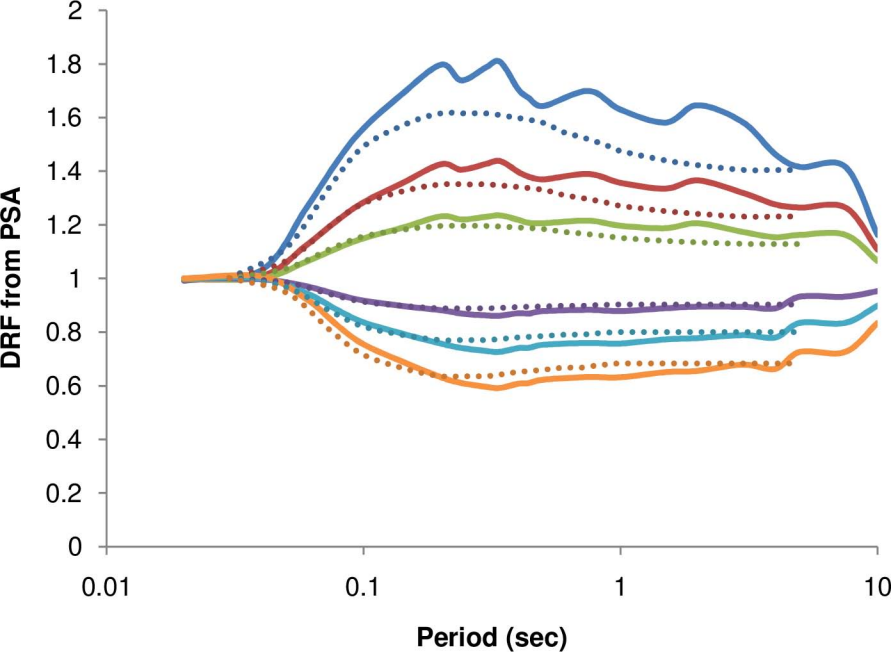

Supplement: S2 Fig — The solid lines represent the proposed model, while the dotted lines represent the model developed by Idriss [11]. (PDF) [file pone.0161137.s002.pdf]
